# Genome-wide genetic structure and differentially selected regions among Landrace, Erhualian, and Meishan pigs using specific-locus amplified fragment sequencing

**DOI:** 10.1038/s41598-017-09969-6

**Published:** 2017-08-30

**Authors:** Zhen Li, Shengjuan Wei, Hejun Li, Keliang Wu, Zhaowei Cai, Dongfeng Li, Wei Wei, Qifa Li, Jie Chen, Honglin Liu, Lifan Zhang

**Affiliations:** 1 College of Animal Science and Technology, Nanjing Agricultural University, Nanjing, 210095 China; 2 Shanghai Animal Disease Control Center, Shanghai, 201103 China; 3 College of Animal Science and Technology, China Agricultural University, Beijing, 100193 China; 4 Laboratory Animal Research Center, Zhejiang Chinese Medical University, Hangzhou, 310053 China

## Abstract

As typical Chinese indigenous pig breeds, Erhualian and Meishan have been widely used to produce new strain or breed in the world. However, the genetic basis of characteristics of these pig breeds is still limited. Moreover, considering cost and output of sequencing, it is necessary to further develop cost-effective method for pig genome screening. To contribute on this issue, we developed a SLAF-seq (specific-locus amplified fragment sequencing) method for pigs and applied it to analyze the genetic difference among Landrace, Erhualian, and Meishan pigs. A total of 453.75 million reads were produced by SLAF-seq. After quality-control, 165,670 SNPs (single nucleotide polymorphisms) were used in further analysis. The results showed that Landrace had distinct genetic relationship compared to Erhualian (*F*_ST_ = 0.5480) and Meishan (*F*_ST_ = 0.5800), respectively, while Erhualian and Meishan held the relatively close genetic relationship (*F*_ST_ = 0.2335). Furthermore, a genome-wide scanning revealed 268 differentially selected regions (DSRs) with 855 genes and 256 DSRs with 347 genes between Landrace and the two Chinese indigenous pig breeds and between Erhualian and Meishan, respectively. This study provides a new cost-effective method for pig genome study and might contribute to a better understanding on the formation mechanism of genetic difference among pigs with different geographical origins.

## Introduction

In 2009, the pig genome was being sequenced and annotated for public utility^1^, which made it possible for analysis and application of genome-wide genetic variants of pig. Shortly afterwards, Illumina developed the PorcineSNP60 Genotyping BeadChip, which included around 60,000 loci by genotyping 158 individuals across multiple pig breeds^2^. Due to the advantages of high density and inexpensive price, the Illumina PorcineSNP60 Genotyping BeadChip has been widely used in multiple research areas. For example, identifying candidate genes associated with economically important traits in different pig populations^3–5^, detecting inbreeding depression of reproductive traits in Iberian pigs^6^, mining genetic diversity and selection signatures in Chinese and Western pig breeds^7^, and discovering population structure of local pig breeds from Russia, Belorussia, Kazakhstan, and Ukraine^8^. These studies greatly enhance our knowledge for understanding the information implied in the pig genome.

Simultaneously, with the rapid development of the next generation sequencing technology, genome resequencing has also been introduced into the pig genome research. i.e., identifying candidate loci related to economically important traits between Korean and European origin breeds^9^, digging signatures of selection in European or Asian domestic pig genome^10, 11^, and measuring coancestry and fitness in Sus cebifrons and Pietrain pigs to design optimal breeding or conservation programs^12^. As a full-scanning approach for discovering all possible genome-wide variants, genome resequencing is considered as a more comprehensive solution for resolving the mystery of the genome. But so far, to some extent, the high costs of sequencing and bioinformatics analysis from genome resequencing have limited its application in population genetics of domestic animals, particularly in some research groups with insufficient funds.

Alternatively, other approaches based on reduced representation genome sequencing strategy to reduce complexity in the genome for resequencing can be applied in pig genome-wide study^13–15^. For example, genome reducing and sequencing (GGRS), which contains more number of loci but similar or lower costs than that of Illumina PorcineSNP60 Genotyping BeadChip, has recently been used to identify selection signatures in Yorkshire and Landrace pigs^16^ and discover the genetic diversity of Chinese indigenous pig breeds of the Taihu Lake region^17^. Meanwhile, another reduced representation genome sequencing, named specific-locus amplified fragment sequencing (SLAF-seq), was also exploited. As a reduced representation library and high-throughput sequencing based approach, SLAF-seq can pre-design the experimental project through bioinformatics and selection of fragments with a specific length in constructing library. More importantly, SLAF-seq has been considered as a high cost-effective way for large-scale genotyping due to its advantages of high genotyping accuracy, high efficiency for marker discovery, low cost, and high capacity for large populations^18^. Therefore, SLAF-seq is widely applied for revealing loci or genes associated with characteristic traits of multiple breeds. i.e., fruit traits in cucumber^19^, tolerance to low-phosphorus stress in soybean^20^, and disease, growth and carcass traits in chicken^21–23^. Obviously, these data support that reduced representation genome sequencing strategy is a convenient and effective way for genome-wide study in plants and animals.

As representatives of Chinese pigs, Erhualian and Meishan are two native pig breeds with high reproductive performance and excellent meat quality such as high litter size and intramuscular fat content. Most recently, many single nucleotide polymorphisms (SNPs) and candidate genes associated with reproductive or meat quality traits in Erhualian pigs have been identified using Illumina PorcineSNP60 Genotyping BeadChip^24, 25^. Additionally, due to high costs, a limited number of pigs were used (usually from 4 to 10 individuals) for genome resequencing and most of the studies classified Erhualian and Meishan as one of domesticated pig breeds rather than separate groups for further analysis^10, 26, 27^. Knowledge of the genetic difference between Erhualian and Meishan pig breeds is lacking. Furthermore, a genome-wide view of possible candidate loci or genes associated with the characteristics of Erhualian and Meishan pigs compared with other distant breeds such as European origin pigs has still not been well investigated. Taken together, for the first time, a SLAF-seq approach for pigs was developed in this study and then was performed to analyze the genetic difference among Landrace, Erhualian, and Meishan pigs, thus providing a cost-effective way for pig genome-wide study and new guidance for understanding the genetic basis of germplasm characteristics of these pig breeds.

## Results

### Development of SLAF markers in the pig genome

In order to exploit SLAF markers, in silico restriction analysis was firstly performed on the current pig reference genome (Sscrofa 10.2). As a result, two restriction enzymes, *Rsa*I and *Hae*III, were used as enzyme combination for developing around 500,000 SLAFs. Finally, a total of 516,733 SLAF tags were predicted using in silico restriction analysis, which showed similar average SLAF distance among different chromosomes (Supplementary Table S1).

### Specific-locus amplified fragment sequencing (SLAF) basics

To obtain the actual SLAF markers used in this study, SLAF-seq was performed in 28 Landrace, 26 Erhualian, and 27 Meishan individuals using the same enzyme combination as in silico restriction analysis. As shown in Table 1 and Supplementary Table S2, a total of 453.75 million reads were obtained from all individuals, which showed that average Q30 and GC content were 87.67% and 44.55%, respectively. Similar to the number of expected SLAFs, 461,261 unique SLAF tags, with average SLAF numbers and sequencing depth as 398,034 (12.74×), 344,471 (6.10×), and 369,160 (6.13×) in Landrace, Erhualian, and Meishan pig populations, respectively, were identified further (Table 1 and Supplementary Table S3). Moreover, compared to the distribution of the expected 516,733 SLAF tags, similar uniformly distribution of 461,261 SLAF tags was also found on different chromosomes (Supplementary Figure S1). In addition, *Oryza sativa indica* was used as a control during sequencing, the results found that the percentage of digestion normally and paired-end mapped reads of control were 91.73% and 90.68%, respectively, indicating that SLAF-seq process was normal and available.

**Table 1:** Characteristics of SLAF-seq among Landrace, Erhualian, and Meishan.

Item	Landrace	Erhualian	Meishan	Average of individuals	*Oryza sativa indica*
Statistics of reads					
Q30 percentage (%)	83.26–87.40	87.03–89.21	87.51–89.89	87.67	88.24
GC percentage (%)	42.93–46.89	44.26–46.76	42.74–45.32	44.55	42.48
Number of reads	7,314,304–11,830,078	2,572,513–5,014,955	3,151,950–5,353,332	5,601,972	1,452,138
Average of reads	9,038,472	3,601,484	3,964,589	—	—
Statistics of SLAFs					
Number of SLAFs	352,574–420,742	316,233–382,547	349,762–389,120	371,217	—
Average of SLAFs	398,034	344,471	369,160	—	—
Average depth	12.74	6.10	6.13	—	—
Statistics of SNPs					
Number of SNPs	1,615,995–2,604,078	1,160,477–1,663,707	1,322,101–1,701,153	1,680,561	—
Heterozygosity (%)	4.81–8.12	5.81–8.45	5.20–7.23	6.51	—
Ratio of Integrity (%)	30.07–48.45	21.59–30.95	24.60–31.65	31.26	—
Average of SNPs	2,176,131	1,302,927	1,530,285	—	—

### Identification of SNPs in Landrace, Erhualian, and Meishan pigs

After genomic mapping and SNP calling, a total of 5,373,997 SNPs were discovered using all individuals (Supplementary Table S4). Moreover, an average of 2,176,131, 1,302,927, and 1,530,285 SNPs were discovered in Landrace, Erhualian, and Meishan pig populations, respectively (Table 1 and Supplementary Table S4). A series of quality control filtering of SNPs were performed to identify 165,670 SNPs used in the further analysis (Supplementary Table S5). Specifically, 161,377 autosomal SNPs were included in linkage disequilibrium and differentially selected region (DSR) analyses, while the 4,293 SNPs on chromosome X were used for DSRs analysis only (Supplementary Figure S2). To reduce the effect from ascertainment bias, a subset of 107,626 autosomal SNPs with minor allele frequency (MAF) ≥ 0.2 was produced and used in genetic structure analysis (Supplementary Figure S2).

### Measuring of linkage disequilibrium by chromosomes

Linkage disequilibrium (r^2^) for pairs of loci was measured by chromosomes in each pig population. As shown in Supplementary Figure S3, the average r^2^ values dropped quickly when physical distances reached 40 kilo base pairs (Kb). With the increasing of physical distance, the similar decreasing trends of the average r^2^ values were observed among three populations. In addition, average r^2^ values of Landrace and Meishan were higher than that of Erhualian.

### Accessing genetic structure, heterozygosity and gene diversity

**Figure 1:**
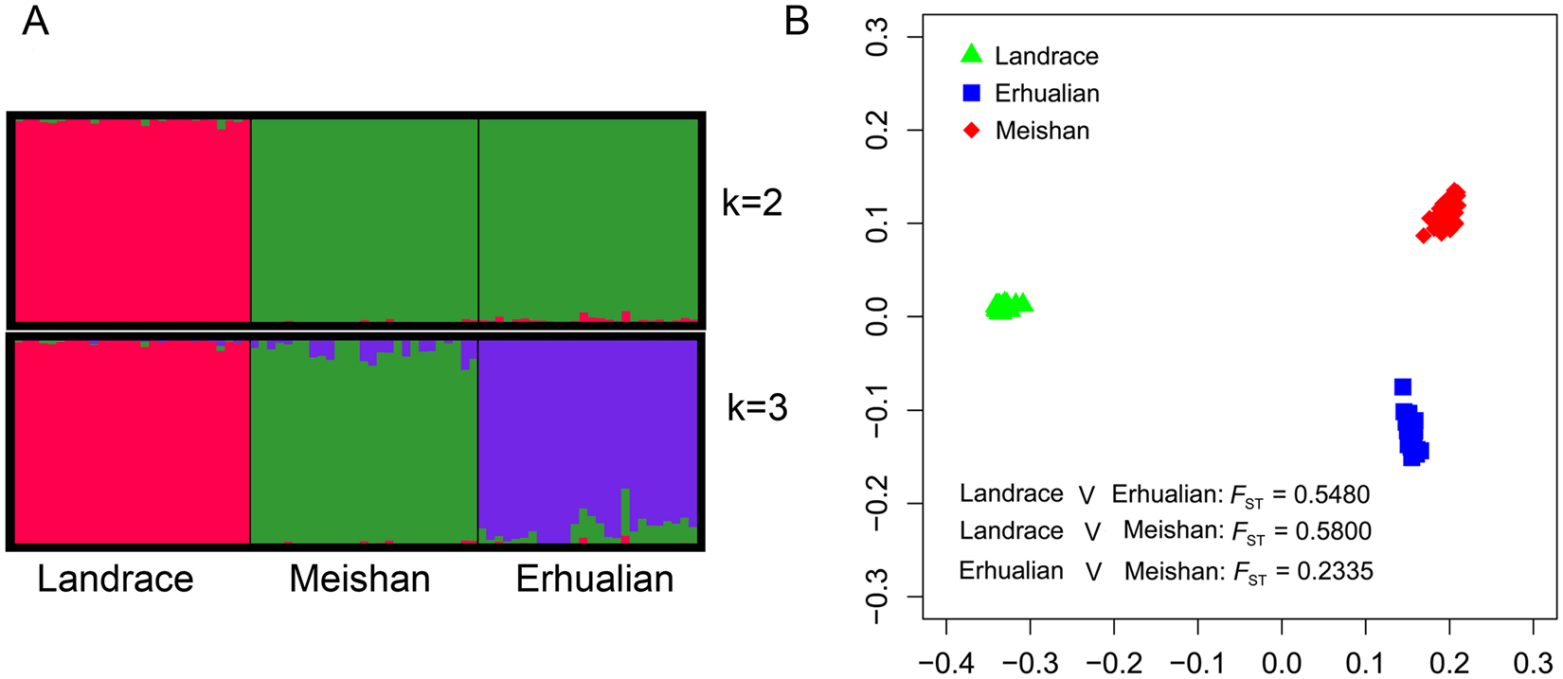
Population structure analysis of the genetic differentiation among Landrace, Erhualian, and Meishan. (**A**) Population structure among Landrace, Erhualian, and Meishan using STRUCTURE software. (**B**) Multidimensional scaling plots among Landrace, Erhualian, and Meishan using PLINK software. *F*_ST_ represents the pair-wise *F*_ST_ between any two pig populations.

As expected, population structure analysis revealed that Landrace and Meishan formed the first two independent populations (K = 2) followed by the Erhualian (K = 3) (Fig. 1A). Moreover, a multidimensional scaling plot clearly showed that Landrace was a distant population compared with Erhualian and Meishan (Fig. 1B). Based on pair-wise estimates of *F*_ST_ described by Weir and Cockerham^28^, the significant genetic differentiation appeared between Landrace and Erhualian (*F*_ST_ = 0.5480), between Landrace and Meishan (*F*_ST_ = 0.5800), and between Erhualian and Meishan (*F*_ST_ = 0.2335), respectively, demonstrating that Erhualian and Meishan hold closer genetic relationship compared to Landrace. As shown in Supplementary Figure S4, observed heterozygosity of Landrace, Erhualian, and Meishan was 0.1956, 0.2410, and 0.2432, respectively, while the gene diversity of Landrace, Erhualian, and Meishan was 0.2276, 0.2952, and 0.2776, respectively. Erhualian and Meishan had the higher observed heterozygosity and gene diversity than that of Landrace.

### Identification of DSRs between Landrace and the two Chinese indigenous pig breeds

**Figure 2:**
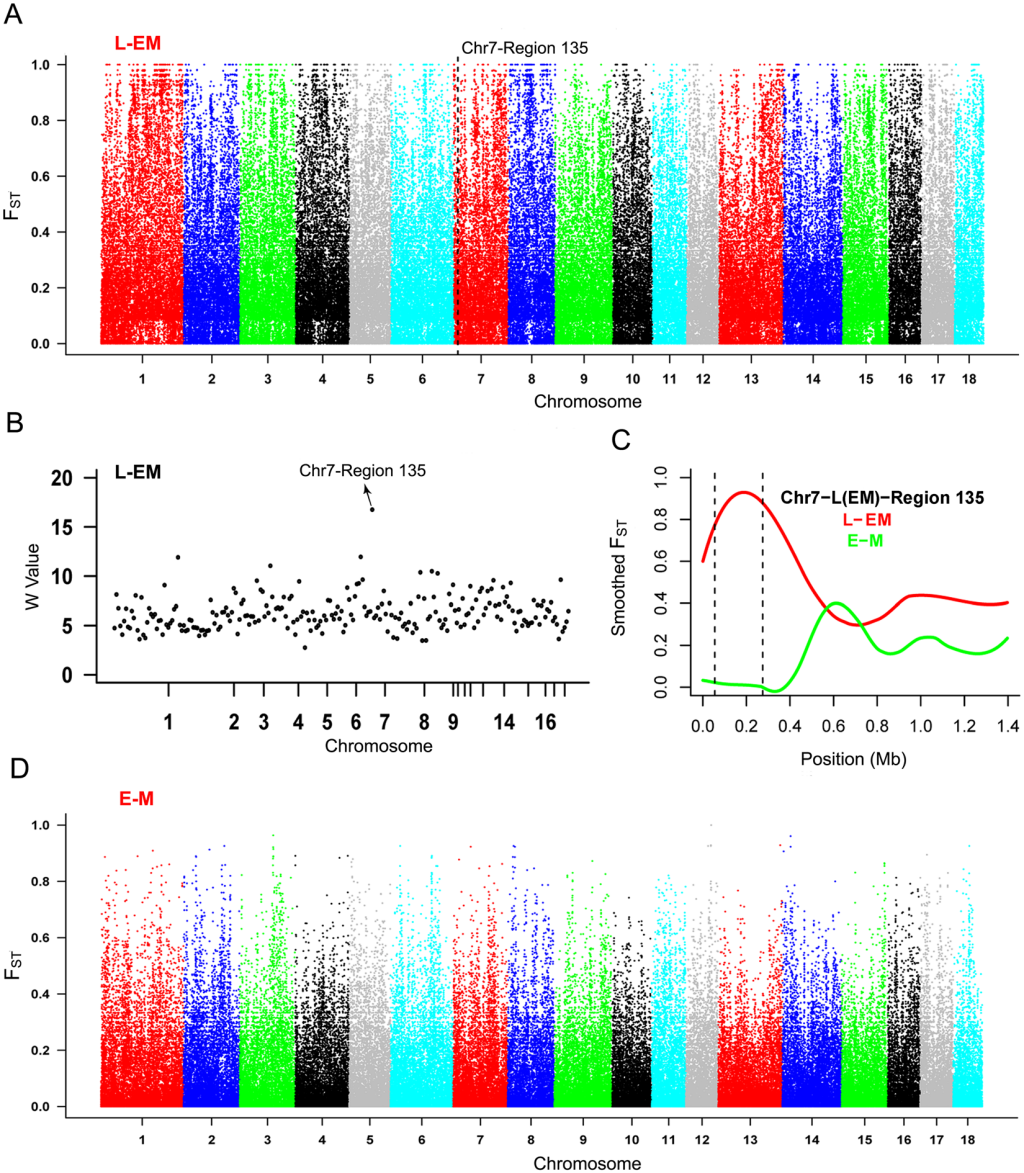
Global distribution of *F*_ST_ among Landrace, Erhualian, and Meishan. (**A**) Global distribution of *F*_ST_ between Landrace and the two Chinese indigenous pig breeds on autosomes 1–18. A representative DSR (region 135 of SSC7) with both the richest top SNPs and the highest W value was indicated. (**B**) The W-statistics was performed for DSRs between Landrace and the two Chinese indigenous pig breeds. (**C**) Smoothed *F*_ST_ showed strong selection signals in region 135 between Landrace and the two Chinese indigenous pig breeds. L-EM represents Landrace-the two Chinese indigenous pig breeds, while E-M means Erhualian-Meishan. (**D**) Global distribution of *F*_ST_ between Erhualian and Meishan on autosomes 1–18.

To obtain the genomic difference among different pig breeds, a SNP-based *F*_ST_ estimation was performed to identify DSRs, which defined regions with shared differentially selection signals across breeds. As these two Chinese indigenous pig breeds have similar origin, Erhualian and Meishan were firstly merged as one population compared with Landrace. As a result, a total of 268 DSRs were identified between Landrace and the two Chinese indigenous pig breeds, which included 237 DSRs in autosomes and 31 DSRs in chromosome X (Supplementary Table S6). The DSR (named region 135) with both the richest top SNPs and the highest W value was located on chromosome 7 (Table 2 and Fig. 2A–C). Moreover, 855 unique known genes, including 511 genes from autosomal DSRs and 344 genes from X chromosome DSRs (Table 2 and Supplementary Table S6), were discovered further, which included many genes associated with economically important traits, i.e., *ESR1* and *KIT*. Furthermore, the gene functional analysis showed that the biological processes between Landrace and the two Chinese indigenous pig breeds were enriched in flavonoid related metabolic process, pigment biosynthetic process, response to hormone, and regulation of developmental process (Supplementary Table S7).

**Table 2:** Top 20 percent of autosomal differentially selected regions (DSRs) among Landrace, Erhualian, and Meishan.

Region	W value	Chr	Position	Top 0.5%	Top 5%	Gene	Candidates
DSRs between Landrace and the two Chinese indigenous pig breeds							
2	8.12	1	16639564..16894373	0	16	1	*ESR1*
12	8.02	1	125860631..126237234	1	13	5	*ALDH1A2,AQP9*
15	7.82	1	157931791..158647002	2	16	8	*NA*
27	9.07	1	245581640..246493427	3	20	6	*SMARCA2*
34	11.89	1	248939813..249341671	11	17	3	*FAM189A2,C1H9orf135*
51	7.56	1	305534056..305560192	4	9	1	*RAPGEF1*
63	8.74	2	132479091..132493356	4	7	1	*CSNK1G3*
64	8.29	2	139541646..139596463	0	9	2	*ACSL6,MEIKIN*
68	7.56	2	153497601..153536938	0	6	1	*PRELID2*
75	9.52	3	40440733..40671307	9	7	25	*SRRM2,TCEB2,BICDL2, FLYWCH1,CCDC64B, LOC100737121,LOC102158577*
77	8.60	3	41967307..42842043	9	4	48	*KCTD5,UNKL,PDPK1,RNPS1*
78	8.41	3	42957871..43071250	4	7	2	*IARS*
82	11.03	3	77090252..77374423	7	15	7	*ARHGAP25,BMP10, APLF*
83	7.87	3	78080175..78118235	2	6	0	*NA*
89	7.86	4	47500407..47599740	2	7	0	*NA*
92	7.69	4	53117484..53787644	3	7	2	*MMP16*
93	7.95	4	55932479..56325201	3	3	3	*CA2,LOC100154871*
97	9.45	4	90369121..90399119	0	17	2	*TBX19,SFT2D2*
122	8.81	6	80274787..80286579	6	2	0	*NA*
123	7.60	6	80425263..80462131	4	3	0	*NA*
126	7.93	6	82230546..82355861	0	10	1	*NA*
127	9.18	6	82692715..82761603	0	9	7	*TXLNA,LOC100739580*
128	9.25	6	82958401..83041048	1	8	4	*ZBTB8A,RBBP4, ZBTB8OS*
129	11.94	6	83940808..84077674	6	14	1	*LOC100523068*
130	9.63	6	84350867..84495848	3	9	1	*C6H1orf94*
135	16.74	7	55136..274807	11	23	1	*EXOC2*
141	8.48	7	60246223..60417535	3	8	3	*ANPEP,ARPIN*
159	7.89	8	55208909..55760237	5	6	7	*LOC102159670*
160	10.37	8	57463293..58027011	1	11	12	*HOPX,THEGL, REST*
162	7.70	8	60649748..62169164	9	4	0	*NA*
166	10.48	8	69657439..69988936	4	16	7	*CENPC,UBA6, LOC100512727*
167	7.86	8	70578162..71233566	9	4	10	*AMTN,UGT2A1,LOC100515222*
169	10.27	8	71666052..72013805	13	4	3	*SLC4A4,LOC100739482*
175	8.18	8	148318210..148486319	7	5	8	*CSN1S2,ODAM, PRR27*
177	9.10	9	44454513..44465001	5	2	1	*DIXDC1*
182	7.72	11	52521631..52714330	3	6	2	*NA*
186	8.96	12	20061666..20149830	2	7	4	*BRCA1*
189	7.58	13	42228668..42395388	0	7	2	*CCDC66,FAM208A*
191	8.41	13	194337969..194393374	0	8	0	*NA*
192	8.76	13	200757712..201353485	0	13	1	*NA*
195	8.46	13	210377658..210418642	0	9	0	*NA*
197	8.70	13	216483385..216515468	6	2	2	*NA*
198	9.56	14	22924788..23026160	0	9	0	*NA*
203	8.86	14	135519785..135551251	0	9	1	*NA*
205	7.91	14	137758835..137835354	3	5	1	*CCDC172*
207	9.29	14	144278853..144330521	0	12	2	*BUB3*
233	9.62	18	43951415..44344272	3	10	2	*BBS9,LOC100738822*
**DSRs between Erhualian and Meishan**							
7	9.63	1	15312287..15312564	4	2	0	*NA*
29	10.25	2	26179507..26497490	3	15	0	*NA*
35	10.89	2	122905147..123003827	3	7	0	*NA*
45	10.22	2	143611291..143611845	1	7	1	*SLC25A48*
46	9.51	3	4355611..4371774	5	1	2	*RADIL,MMD2*
52	9.87	3	90528360..90824884	3	3	8	*CCDC104,CLHC1, SMEK2, MTIF2*
56	11.87	3	102297392..102494238	4	5	4	*PPM1B,PREPL, SLC3A1*
58	9.91	3	102678119..102743126	4	2	1	*LRPPRC*
59	9.83	3	106283461..106442746	1	8	1	*SLC8A1*
61	9.97	3	113055246..113120757	4	1	1	*LOC100626840*
64	10.87	3	115856309..116048881	6	7	4	*YPEL5*
67	8.83	4	18251390..18266000	0	6	0	*NA*
75	9.11	5	5199728..5220416	3	4	2	*SGSM3,MKL1*
78	10.20	5	19478109..19576660	4	8	1	*NA*
98	10.37	6	102930166..103055274	5	1	2	*ZNF521*
99	15.01	6	103154776..103528819	14	0	2	*NA*
100	12.80	6	103780912..104046778	9	3	4	*PSMA8,KCTD1,TAF4B*
104	11.46	6	124785239..124938047	6	4	2	*PTGFR*
110	10.20	7	34828000..34927976	3	1	2	*GRM4*
111	9.08	7	39781167..39790945	3	3	0	*NA*
113	12.53	7	91745010..91808309	6	5	0	*NA*
117	10.97	7	105180004..105206797	3	3	2	*IFT43,TGFB3*
118	9.18	8	10911848..10912137	4	0	2	*PROM1,LOC102160409*
121	9.71	8	13697594..14004707	3	2	0	*NA*
134	14.91	9	40038695..40228268	12	2	8	*CWF19L2,ELMOD1*, *ALKBH8,LOC100626234*
138	8.79	9	95612266..95753391	0	7	0	*NA*
139	12.75	9	105189739..105220813	7	0	0	*NA*
143	10.80	9	142936175..143029098	2	10	0	*NA*
146	10.06	10	3770676..3770960	1	5	0	*NA*
147	9.17	10	31350537..31364218	2	2	1	*C10H9orf3*
164	8.86	11	50583012..50964515	2	17	2	*KLF12,LOC100622863*
177	9.00	12	46196899..46237519	4	6	0	*NA*
180	14.14	12	51152923..51247795	8	0	2	*NA*
182	9.99	13	12498982..12503925	0	7	1	*THRB*
183	11.92	13	13052089..13079543	0	9	0	*NA*
185	15.35	13	189288665..189354590	1	10	1	*LIPI*
186	12.39	13	190678701..190700337	0	13	0	*NA*
188	10.51	13	216737510..216756384	2	3	0	*NA*
192	16.19	14	16460290..16486569	10	5	0	*NA*
195	11.06	14	21329899..21678452	5	8	4	*CLCN3,NEK1*
201	10.68	14	47963670..48581378	1	11	5	*MN1,PITPNB,TTC28*
202	12.12	14	48900016..49663109	2	15	17	*EWSR1,KREMEN1,NIPSP1, RHBDD3*
218	10.39	15	152011389..152018640	2	2	1	*UBE2F*
223	8.72	16	24195627..24417936	2	15	1	*NA*
230	9.00	16	82918895..82946838	3	2	0	*NA*
235	8.67	17	11918899..11919532	0	7	0	*NA*
236	9.23	17	15112581..15187900	3	3	3	*TMEM230,CDS2*
237	9.47	17	15425824..15480579	1	8	1	*GPCPD1*
240	10.06	17	47494168..47511555	4	1	1	*PPP1R16B*
252	9.81	18	41156162..41171465	2	6	2	*AOAH*

Note: The candidate genes were given within the SNPs of top SNP (0.5% or 5%) for each region.

### DSRs associated with female characters between Landrace and the two Chinese indigenous pig breeds

In order to further investigate DSRs or genes related to female characters, a female based genome-wide screening was performed between Landrace and the two Chinese indigenous pig breeds. A dataset with MAF ≥ 0.1 containing 175,952 SNPs from autosomes and 8,320 SNPs from chromosome X was used in this analysis. Based on the SNP *F*_ST_ estimates, 241 DSRs in autosomes and 60 DSRs in chromosome X were revealed. Further examination of these DSRs identified 443 unique known genes from autosomes and 327 genes from chromosome X (Supplementary Table S8). GO terms of these genes from DSRs were involved in chromatin organization, odontogenesis, animal organ development, and reproductive system development (Supplementary Table S9).

### Characterization of DSRs between Erhualian and Meishan

A genome-wide distribution of *F*_ST_ between Erhualian and Meishan on autosomes is shown in Fig. 2D. Between these two pig breeds, 256 DSRs, including 252 DSRs in autosomes and 4 DSRs in chromosome X, were discovered. Moreover, 341 unique known genes from autosomal DSRs and 6 genes from X chromosome DSRs were discovered further (Supplementary Table S10). The gene enrichment analysis found that DSRs between Erhualian and Meishan were enriched in sodium-independent organic anion transport, glycerophospholipid metabolic process, and phosphorus or phospholipid related metabolic process (Supplementary Table S11).

## Discussion

In the present study, we used SLAF-seq to analyze the genetic structure and differentially selected regions among Landrace, Erhualian, and Meishan pigs. By using over 160,000 SNPs developed in this study, our results clearly indicated that there was a closer genetic relationship between Erhualian and Meishan compared with the genetic relationship between Landrace and Erhualian or Meishan. Moreover, 268 DSRs including 855 genes were discovered between Landrace and the two Chinese indigenous pig breeds, while 256 DSRs including 347 genes were found between Erhualian and Meishan. As such, our data provide a comprehensive view of the genome-wide basis for genetic difference among these pig breeds.

Basically, as a high-resolution method for genome-wide genotyping, SLAF-seq is considered as an enhanced reduced representation sequencing and possesses several remarkable features^18^, i.e., accurate genotyping by depth paired-end sequencing, reduced sequencing costs, pre-design strategy for fragmentation efficiency, and double barcode system for large populations. In this study, a total of 516,733 SLAFs from the pig reference genome (Sscrofa 10.2) were predicted using *Rsa*I and *Hae*III enzyme combination. With more than 6 × sequencing depth but below $80 cost for each individual, 5,373,997 SNPs were discovered from Landrace, Erhualian, and Meishan pig populations by SLAF-seq. After filtering, 165,670 SNPs were available for analyzing the genetic difference among these pig populations, which would have an average inter-marker distance of approximately 17 Kb and several multiples of the number of SNPs from Illumina PorcineSNP60 Genotyping BeadChip. Compared with the published pig GGRS, which contained 34,789–105,550 available SNPs for different objective analysis^14, 17^, our SLAF-seq method might provide more genomic variation information. More importantly, our data explored the genetic difference between Landrace and the two Chinese pig breeds using reduced representation sequencing strategy, which has not been well studied by GGRS. Considering the huge difference in genome variation between European and Chinese pig breeds, our data demonstrate that SLAF-seq is a powerful approach and has great potential for further studies in more breeds. Therefore, the SLAF-seq approach could be considered as a more competitive choice for pig genome study.

It is well known that Landrace was originally developed in Denmark, while Erhualian and Meishan were domesticated in China, implying that Landrace was as a geographically distant breed relative to Erhualian and Meishan. As expected, both population structure and cluster analysis showed that Landrace was genetically distant from two Chinese indigenous pig breeds (Fig. 1). Moreover, pair-wise *F*_ST_ estimation supported Landrace had distant genetic relationship compared with two Chinese indigenous pig breeds (*F*_ST_ = 0.5480 for Erhualian and *F*_ST_ = 0.5800 for Meishan), demonstrating that there was a great genetic differentiation between Landrace and two Chinese indigenous pig breeds. These results were consistent with the data from other genetic markers such as mitochondrial DNA and microsatellite markers^29, 30^, which showed European and Chinese pig breeds exist long genetic distances. In particular, we found there was close but significant genetic differentiation (*F*_ST_ = 0.2335) between Erhualian and Meishan. Actually, a significant genetic differentiation between Erhualian and Meishan had been observed using GGRS methods^17^. Therefore, our data provide a further evidence that Erhualian and Meishan should be two pig breeds, although they had been classified as the same pig breed named Taihu pigs in the annals of history as both pig breeds lived in Taihu Lake regions, eastern of China. Additionally, higher observed heterozygosity and gene diversity were found in these two Chinese indigenous pig breeds compared with Landrace, implying that these two Chinese indigenous pig breeds had more abundant genetic diversity. This is not surprising because Chinese indigenous breeds had more genetic diversity than that of European pig breeds^30^. Accordingly, this study provides a global view of the genetic structure and relationship among these three pig populations.

Generally speaking, Erhualian and Meishan are two of the most prolific pig breeds known in the world^24, 31, 32^ and have been mainly considered as the maternal line for synthetic strain selection. Meanwhile, these two pig breeds are used to build specific populations for identifying candidate genes or QTLs (quantitative trait loci) related to reproductive traits, i.e., Iberian x Meishan F2 population^33^, Large White × Meishan cross gilts^34^, and Duroc x Erhualian resource population^35^. Intuitively, compared to Landrace, a major of the maternal sources in European pig breeds, Erhualian and Meishan pigs possess the better farrowing capacity, which results in far more litter size^36, 37^. To further identify the candidates related to female reproductive traits, a female based dataset was used at the same time. Compared with the candidate genes from all individuals, a total of 502 genes from female based DSRs were found overlapping (Supplementary Tables S6 and S8). Many important genes related to female reproductive traits such as *ESR1*, *RBBP4*, and *BRCA1*, were discovered in both DSR datasets. In the previous study, as a key gene for mediating estrogen, *ESR1* with its *Pvu*II site polymorphism had been shown to be associated with litter size in different pig populations, i.e., Meishan synthetic line^38^, Landrace population^39^, a population of Erhualian and Xiang pig breeds^40^, and Large White x Meishan F2 crossbred gilts^41^. However, other studies did not find significant associations of *ESR1* with litter size in a synthetic line of Duroc and Large White origin or a Meishan x Large White F2 population^42, 43^, demonstrating that the role of *ESR1* in sow prolificacy among different pig populations was still controversial. Therefore our data might provide additional information for interpreting the difference in litter size between Landrace and the two Chinese indigenous pig breeds. *RBBP4*, a biomarker of oocyte competence, had different expression levels between *in vivo*- and *in vitro*-matured oocytes in bovine^44^ and could affect bipolar spindle assembly by regulating histone deacetylation during oocyte maturation in mouse^45^. However, little is known about the function of *RBBP4* in pig reproductive process. Our data suggest that *RBBP4* might have an important role in sow reproductive performance, but confirmation of this assumption warrants further study. Interestingly, BRCA1, a key DNA repair protein, was identified in DSRs. Previously, genetic variants of *BRCA1* were mainly related to breast or ovarian related diseases in human and cattle^46, 47^. Besides, reducing numbers of primordial follicles and simultaneously increasing DNA double-strand break repair with age were observed in BRCA1-deficient mice compared to the wild-type mice, showing that BRCA1-deficient led to ovarian aging^48, 49^. In women, *BRCA1* mutation produced lower numbers of eggs compared with control^50^. These data indicated *BRCA1* might play a critical role in regulating ovarian development and the number of eggs. Hence our results might give us a novel clue for its role in pig reproductive performance.

Furthermore, a DSR with both the richest top SNPs and the highest W value in autosomes was found on *Sus scrofa* chromosome 7 (SSC7, 0.05 to 0.27 Mb) between Landrace and the two Chinese indigenous pig breeds (Fig. 2, Table 2 and Supplementary Table S6). Interestingly, EXOC2, a component of the exocyst complex, was found in this region. Previous studies indicated that *EXOC2* played a role in filopodia formation by binding the GTPase RalA in Swiss 3T3 cells and mediated vesicle trafficking by interacting with the Rho guanine nucleotide exchange factor (GEF)-H1 in HeLa cells^51, 52^. Additionally, genetic variants of *EXOC2* or adjacent *EXOC2* had been considered as contributing to pigmentary traits such as hair color, skin pigmentation and tanning ability in Europeans as well as vitamin D status in the Caucasian population^53–57^. In this study, Landrace has white hair color and skin, while Erhualian and Meishan have colored hair and skin^37^, indicating that there is great difference in pigmentary traits between Landrace and the two Chinese indigenous pig breeds. Our data suggest that *EXOC2* might be a good pigmentation trait candidate of pigs. However, the biological mechanism of *EXOC2* has not been well characterized in regulating human pigmentation. Moreover, little is known about the role of *EXOC2* in pigs. Therefore, confirmation and elucidation the role of *EXOC2* in pigmentation trait in pigs requires further investigation. Actually, another key well-documented pigmentary gene, *KIT* was also discovered in DSRs (Supplementary Table S6). In the past two decades, *KIT* had been deeply investigated for its mutation associated with coat color of the dominant white in pigs^58–60^. Moreover, as a colored breed, Meishan had been found to have different *KIT* allele distinguish from white breed such as Landrace and Large White^58, 60^. Obviously, our data provide further evidence for the role of *KIT* in pigmentary traits. More interestingly, we discovered some genes associated with growth or meat quality traits in DSRs, i.e., *ACSL6*, *RAPGEF1*, *BMPER*, and *KDR* (Table 2 and Supplementary Table S6). *ACSL6*, a member of the long-chain acyl-CoA synthetase gene family for catalyzing the formation of acyl-CoA from fatty acids, ATP, and CoA, was shown to be associated with dry matter intake and metabolic mid-test body weight in cattle^61^. RAPGEF1, a guanine nucleotide releasing protein, was discovered to play an important role in skeletal muscle differentiation^62^ and was significantly associated with type 2 diabetes in the Korean population and Finns^63, 64^. These studies demonstrated that *ACSL6* and *RAPGEF1* might have important function in body growth or development of skeletal muscle and fat. Hence our results might provide new ideas for their function in affecting pig growth and development. In addition, *BMPER* and *KDR*, two key genes in the development of angiogenesis and vasculogenesis^65, 66^, were identified in DSRs. It is not surprising because our previous study had found genetic variants in the promoters of *BMPER* and *KDR* affected intramuscular fat deposition in Erhualian pigs^67, 68^. Moreover, *BMPER* was found to be associated with rump length and body size in Qinchuan cattle population^69^. Here our data suggest that *BMPER* and *KDR* might contribute to the regulation of pig growth and meat quality, but additional studies are needed to further confirm this speculation. In a word, our results provide a comprehensive clue for discovering the genetic difference between Landrace and the two Chinese indigenous pig breeds.

As described above, Erhualian and Meishan are two famous pig breeds with the typical characters of Chinese indigenous breeds such as high productive performance and good meat quality. However, as two different pig breeds, some characteristics, i.e., head wrinkles and colors of limbs and nose^37^, are very different between these two pig breeds. In this study, the genetic differentiation (*F*_ST_ = 0.2335) between Erhualian and Meishan was observed, further confirming that there exists a significant genetic difference between these two pig breeds. But until now, only variants from single gene such as *FUT1* have been used to compare the difference in gene frequencies between these two pig breeds^70^. In a genome-wide level, the genetic difference between these two pig breeds is still largely unknown. Our present study identified 256 DSRs containing 347 genes between Erhualian and Meishan. Importantly, GO terms associated with these genes were enriched in sodium-independent organic anion transport, glycerophospholipid metabolic process, and phospholipid related metabolic process. Some important candidates, i.e., *SLCO3A1* in sodium-independent organic anion transport, *CDS2*, *CRLS1*, and *MTMR7* in glycerophospholipid metabolic process, and *SPTLC1*, *SGMS2*, and *CRLS1* in phospholipid related metabolic process, were discovered from enrichment analysis. The difference of the biological processes between these two pig breeds might result from a long-term artificial selection based on different breeding objectives, but further investigation of the cause of breed difference is required.

In summary, a SLAF-seq approach for pigs was developed to reveal the genetic structure and relationship among Landrace, Erhualian, and Meishan pigs. Meanwhile, DSRs including hundreds of genes between Landrace and the two Chinese indigenous pig breeds and between Erhualian and Meishan were identified further. Consequently, our study not only provides a cost-effective approach for pig genome-wide screening, but also establishes the genetic basis for further investigating important gene functions from DSRs among these pig breeds in future research.

## Materials and Methods

### Animal

Blood or ear tissue samples were collected from 28 Landrace (8 male and 20 female), 26 Erhualian (8 male and 18 female), and 27 Meishan (Small Meishan, 8 male and 19 female) at the Xiang Xin livestock and poultry Co., Ltd (Shanghai), Changzhou Erhualian production cooperation (Jiangsu), and Taicang pig breeding farm (Jiangsu), respectively. Total DNA was extracted by the phenol-chloroform extraction method, with concentration and purity measured using the Nanodrop^TM^ 2000 spectrophotometer (Thermo Scientific, Waltham, MA, USA) and electrophoresis. All animal care and handling procedures were approved by the Animal Ethics Committee at Nanjing Agricultural University, China. All methods were performed in accordance with the guidelines and regulations of the Animal Ethics Committee at Nanjing Agricultural University.

### SLAF library construction and sequencing

A simulated restriction enzyme digestion was carried out on the current pig genome (Sscrofa 10.2) to identify expected SLAF yield, avoid repetitive SLAFs, and obtain the relatively uniform distribution of restriction fragments in the genome. As a result, genomic DNA was digested with *Rsa*I and *Hae*III restriction enzyme combination. Meanwhile, in order to assess the experimental procedure, *Oryza sativa indica* (http://rapdb.dna.affrc.go.jp/) was used as a control for evaluating the effectiveness of enzyme digestion and paired-end mapped reads. In brief, SLAF library construction and sequencing for each individual was conducted as described previously^18^ with slight modifications: DNA fragments of 314–364 base pair (bp) were selected as SLAFs and used for paired-end sequencing by Illumina HiSeq 2500 system (Illumina, Inc., San Diego, CA, USA) at Beijing Biomarker Technologies Corporation.

### Genome mapping, SNP calling and filtering

Raw paired-end reads were mapped to the pig reference genome (Sscrofa 10.2) using BWA software^71^. In general, SLAF groups produced by reads were mapped to the same position. If an accession was only partly digested by the restriction enzymes, reads mapped to the reference genome should have overlaps with two SLAF tags and were assigned to both of the SLAF tags in the same accession. SNP calling was performed by both GATK and samtools analysis^72, 73^, and a locus was defined as a SNP if it was simultaneously called from these two packages. Filtering high-quality SNPs was conducted by PLINK v1.07^74^. The analysis of Hardy-Weinberg Equilibrium (HWE) for SNPs from autosomes and chromosome X was performed by Pedstats tools and HWE package for R^75, 76^, respectively.

### Population genetic basics analysis

P﻿opulation structure analysis was performed using STRUCTURE with 10,000 iterations via the correlated allele model^77^, and then plotted by DISTRUCT software^78^. Linkage disequilibrium analysis was computed between each marker pair within each breed separately using Haploview 4.1^79^. The population relatedness of pair-wise *F*_ST_ and gene diversity were calculated using the HIERFSTAT package for R. Observed heterozygosity and IBS matrix of distance were estimated by PLINK v1.07^74^, and then multidimensional scaling (MDS) analysis of autosomal SNPs was determined by R 3.2.4.

### Detection of differentially selected regions (DSRs)

The DSR algorithm was carried out as described previously^3, 80^ with slight modifications: 1) raw values were ranked; 2) Fisher’s exact test was executed in R 3.2.4 to compare the allele frequencies between Landrace and the two Chinese indigenous pig breeds and between Erhualian and Meishan, respectively. SNPs with *P* values < 0.05 were considered as statistically significant after Bonferroni correction; 3) *F*_ST_ of SNP was estimated using the model proposed by Nicholson *et al*.^81^ and Flori *et al*.^82^, and then the significant SNPs with 0.5% or 5% highest *F*_ST_ values were selected as the top significant SNPs; 4) by placing the top significant SNPs (0.5%) in the center of a DSR, adjacent SNPs were collected to determine the region boundaries until more than two consecutive SNPs were not in the top significant 5% threshold. The overlapped DSRs were combined as the same DSR. When a region contained more than five SNPs from the top significant SNPs (5%) but without top significant SNPs (0.5%), we also considered it as a DSR in our study. In addition, the W-statistics was used to identify the relative importance of DSRs^83^ and smoothed *F*_ST_ values were obtained by a local variable bandwidth kernel estimator^84^.

### Gene functional enrichment analysis

Genes in these DSRs were identified using the Sscrofa 10.2 assembly (www.animalgenome.org/cgi-bin/gbrowse/pig) and NCBI database. The functional enrichment analysis of target genes was performed using Panther bioinformatics resources (www.pantherdb.org). Terms with *P* values less than 0.01 and the enrichment value more than 1 were selected as significant or enriched terms.

### Data accessibility

The datasets have been submitted to the SRA database of NCBI (accession number SRP090907).

## Electronic supplementary material

Supplementary Figures

Dataset 1

Dataset 2

Dataset 3

Dataset 4

Dataset 5

Dataset 6

Dataset 7

Dataset 8

Dataset 9

Dataset 10

Dataset 11
