# Supplementary material for: Genome-wide genetic structure and differentially selected regions among Landrace, Erhualian, and Meishan pigs using specific-locus amplified fragment sequencing
Source: Sci Rep. 2017 Aug 30;7:10063. doi: 10.1038/s41598-017-09969-6 (PMC5577042; doi:10.1038/s41598-017-09969-6)
Supplement: Supplementary file 1 — Supplementary Figures [file 41598_2017_9969_MOESM1_ESM.pdf]

Genome-wide genetic structure and differentially selected regions among Landrace, Erhualian, and Meishan pigs using specific-locus amplified fragment sequencing

Zhen Li[1], Shengjuan Wei[1], Hejun Li[2], Keliang Wu[3], Zhaowei Cai[4], Dongfeng Li[1], Wei Wei[1], Qifa Li[1], Jie Chen[1], Honglin Liu[1] & Lifan Zhang[1]

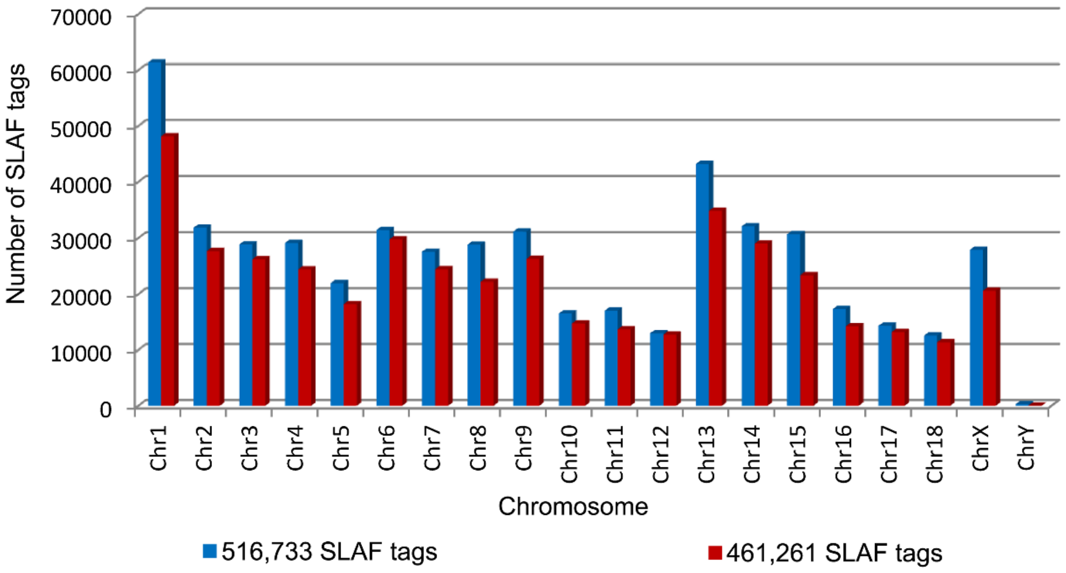

Supplementary Figure S1. Distributions of SLAF tags on different chromosomes

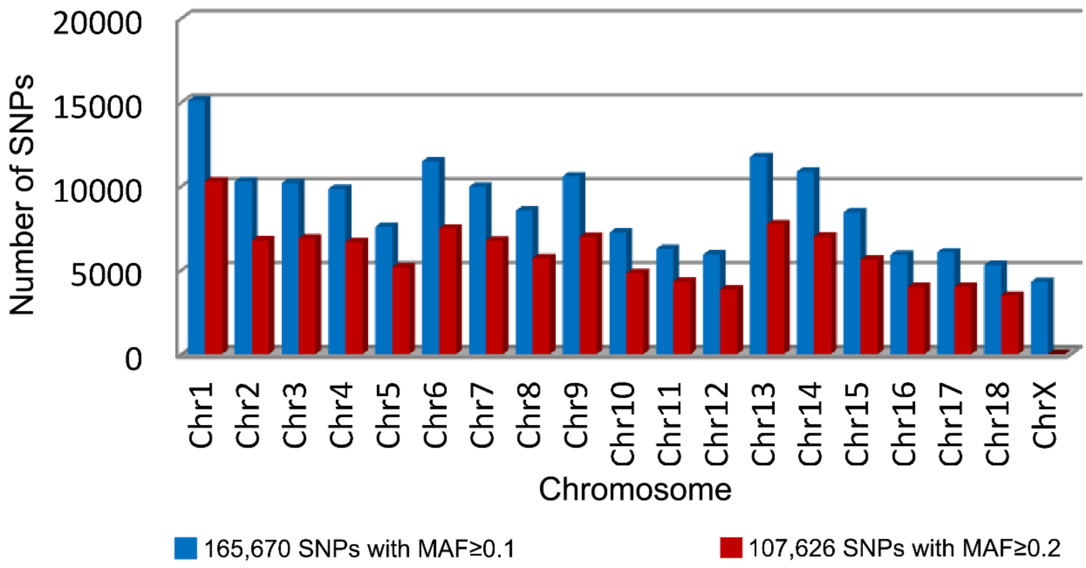

Supplementary Figure S2. Distributions of SNPs on different chromosomes

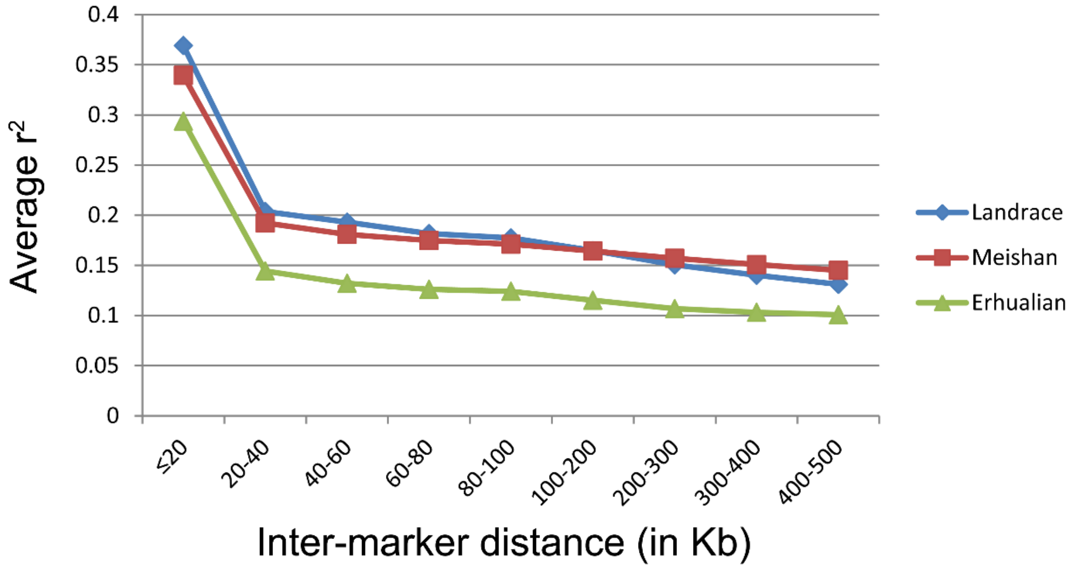

Supplementary Figure S3. Linkage disequilibrium analysis in different pig breeds

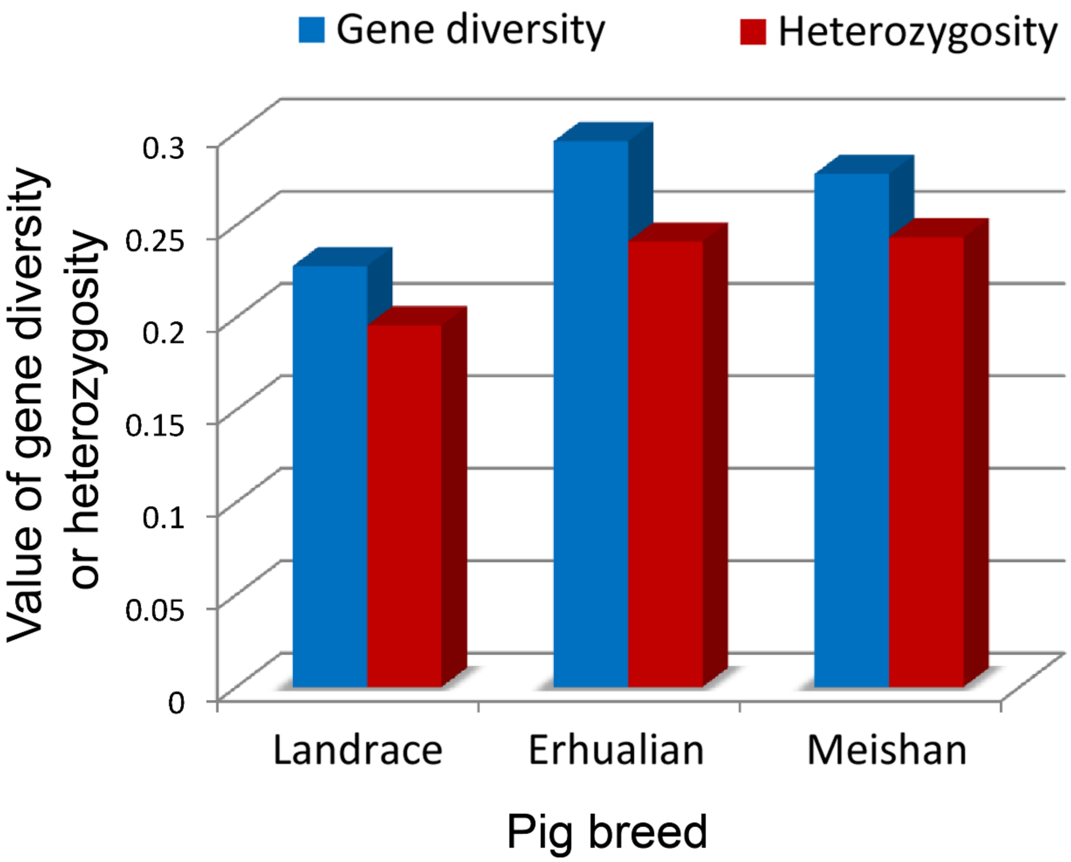

Supplementary Figure S4. Gene diversity and heterozygosity analysis in different pig breeds
